# Clinical performance of short fiber-reinforced composite resin restoration in large posterior cavities: a systematic review and meta-analysis

**DOI:** 10.1038/s41598-025-31441-z

**Published:** 2025-12-22

**Authors:** Kareem Hamdi, Ahmed Elsebaai, Youniss Saleh Harp, Hamdi H. Hamama

**Affiliations:** 1 Conservative Dentistry Department, Faculty of Dentistry, Zagazig University, Zagazig, Egypt; 2 Pediatric Dentistry Department, Faculty of Oral and Dental Medicine, Delta University for Science and Technology, Gamasa, Egypt; 3 Conservative Dentistry Department, Faculty of Dentistry, Mansoura University, Mansoura, Egypt; 4 Faculty of Oral and Dental Medicine, Alsalam University, Tanta, Egypt; 5 Faculty of Oral and Dental Medicine, New Mansoura University, New Mansoura, Egypt; 6 Faculty of Oral and Dental Medicine, Alsalam University, Tanta, Egypt

**Keywords:** Short-fiber reinforced composite resin restorations, Fiber-reinforced composite resin restoration, Composite resin restoration, Conventional composite resin restoration, Posterior teeth, Clinical performance, Systematic review, Diseases, Health care, Medical research

## Abstract

Restoring large posterior vital-teeth cavities remains a clinical challenge. This systematic review and meta-analysis aimed to compare the clinical performance of short fiber-reinforced composites (SFRCs) versus conventional resin-based composites (RBCs) in such cavities. PubMed, Web of Science, and Scopus were searched, identifying 1,632 records. Five randomized controlled trials met inclusion criteria for qualitative synthesis, and four were included in the meta-analysis. Risk of bias was assessed using Cochrane RoB 2.0, and the certainty of evidence was rated via GRADE. Meta-analysis showed no statistically significant difference between SFRCs and conventional RBCs across different clinical parameters following the USPHS criteria. Sensitivity analyses addressed heterogeneity in color match (RR = 0.77; 95% CI 0.57–1.03; *P* = 0.08; I² = 80%) and marginal adaptation (RR = 1.10; 95% CI 0.91–1.33; *P* = 0.32; I² = 70%). However, sensitivity analysis suggested a possible advantage for RBCs in terms of color match (RR = 0.68; 95% CI 0.55–0.83; *P* = 0.0001), these findings are based on a limited number of studies and should be interpreted cautiously. Overall, the certainty of evidence was low, with two trials judged at high risk of bias, two at low risk, and one with some concerns. Current low-certainty evidence suggests that SFRCs perform comparably to conventional RBCs in large posterior cavities. However, given the limited number of trials and variable study quality, these results should be interpreted cautiously. Further high-quality, long-term RCTs are needed to confirm these findings.

**Supplementary Information:**

The online version contains supplementary material available at 10.1038/s41598-025-31441-z.

## Introduction

Recently, resin-based composite restorations (RBCs) have gained popularity due to their alignment with the minimally invasive philosophy, allowing clinicians to avoid unnecessary extensive cavity preparations compared to amalgam restorations^1^. Several attempts were implemented in packable resin composite matrix to enhance their mechanical properties, particularly in extensive posterior cavities^2^. Furthermore, modifications in dentin adhesives and bonding protocols have positioned RBCs as the preferred tooth-colored material, demonstrating satisfactory clinical performance^3^. Nevertheless, a considerable failure rate is still reportable in dental literature with RBCs which often ascribed to secondary caries and bulk fracture^4–7^ The development of secondary caries is closely linked to the inherent polymerization shrinkage characteristic of dental composite restorative materials. Although many technologies were conducted to control polymerization stresses, the volumetric shrinkage remains one of the major drawbacks of the material^8,9^. At level of fracture, it was reported that high failure rates are associated with large cavities, where including extra surface increase the failure risk by 30–40%^10^. Consequently, tooth strengthening remains the inevitable goal for scientific community.

The term ‘biomimetics’ has recently garnered significant attention. Within dental literature, it refers to the replication of natural biological strategies and processes^11^. One of the smart attempts to strength the teeth with extensive cavities and overcome the aforementioned drawbacks is placing fiber-reinforced composite (FRC) in these cavities as dentin substitute. It has been reported that such fibers could regulate and transfer polymerization stresses from polymer matrix a long fibers’ orientation. Moreover, it has been reported that such fibers can minimize or block crack propagation^12^. In this regard, FRC acts as a biomimetic restoration mimicking biologic collagen fibers of tooth dentin.

The effectiveness of fiber-reinforced composite totally depends on different parameters, including the number of fibers in the organic matrix, their type, form, orientation, critical length, and the adhesion of the fibers to the polymer matrix^13^. In this regard, ever-X posterior (GC Corporation, Japan), a short fiber-reinforced composite (SFRC) introduced in 2013 containing short, randomly oriented, discontinuous E-glass fibers for restoring extensive cavities and endodontically treated teeth. A review of the currently available literature revealed that numerous systematic reviews of in vitro studies have evaluated the mechanical and physical properties of SFRC; however, none has assessed its clinical performance^14–16^. Accordingly, the current systematic review has been designed to elucidate the clinical behaviour of SFRCs and address the research question “Are short-fiber reinforced composites clinically efficient restorations in posterior large cavities?”

## Materials and methods

In compliance with the PRISMA (Preferred Reporting Items for Systematic Reviews and Meta-Analyses) guidelines^17^, the present systematic review was registered in PROSPERO (International Prospective Register of Systematic Reviews) under the registration number (CRD42025646345).

### Eligibility criteria

The current systematic review included the clinical studies that evaluated the clinical performance or behaviour of SFRC in posterior teeth. Accordingly, the inclusion criteria were based on PICOS methodology^17^: Population (P): participants with vital permanent posterior teeth (molars or premolars) that required restoration due to carious lesions or replacement of defective restorations, Intervention (I): SFRC in large posterior cavities, defined as Class II or MOD cavities involving two or more surfaces, or involving a cavity depth greater than half of the dentin thickness, as reported by the authors. When cavity dimensions were not explicitly reported, we included studies in which the majority of restored teeth were described as having extensive or large cavities based on clinical descriptions, photographs, or study definitions. Studies restoring only small or minimal cavities were excluded, Comparison (C): conventional direct / indirect RBCs, Outcome (O): durability of the restorations, Study design (S): randomized clinical trial with parallel or split mouth design (RCTs) with at least 12 months follow-up. Studies conducted in languages other than English were also deemed eligible for inclusion. The exclusion criteria included review articles (literature, systematic, scoping, and umbrella reviews), letters to the editor, case reports, laboratory studies, and clinical studies that used materials other than composite resin restorations as comparators.

### Information and search strategy

A comprehensive online search of the published literature was conducted across PubMed (including MEDLINE), Scopus, and Web of Science databases, covering the period from 2013 to January 2025. The literature search was initiated from 2013, the year in which SFRCs were first introduced to the dental market. Detailed information on the search strategy, including keywords and Boolean operators used for each database, is provided in supplementary file 1. A reference manager software (EndNote X9^®^ Basic-Thomson Reuters, New York, USA) was used to identify and remove duplicate hits.

### Data extraction

Data extraction was performed in two separate phases. In the first phase, the titles and abstracts of all studies identified in the electronic database search were independently screened and assessed by two reviewers (K.H. and Y.S.). While, in the second phase, the same reviewers provided a full text screening of relevant studies independently and then collected the key information from the studies. The same reviewers also paid attention to include any intriguing papers that might have been overlooked throughout the search by looking through the references in that pertinent research. Finaly, the selection of relevant articles was obviously depending on full-text assessment. In case of disagreement in paper selection between the same reviewers, third reviewer (H.H) was invited to solve this debate.

### Data items

Specific data items were identified and collected from each relevant study including study name, year of publication, sample size, type of study, number of patients, average age of patients, used composite resin restorations in control and experimental groups, method of substrate etching, type of used adhesive, number of restored teeth, type of restored teeth, composite resin placement technique, follow-up period, and evaluation criteria. Data collection and tabulation was performed by the same reviewers after full-text assessment and presented in Table 1.

### Risk of bias assessment

The risk of bias in the included studies was independently assessed by the same reviewers, following the guidelines outlined in the Cochrane Handbook for Systematic Reviews of Interventions (RoB-2)^18^. Six key domains were evaluated: randomization procedure, deviations from interventions, missing outcome data, measurement of the outcome, selective reporting of results, and other potential sources of bias. Studies were classified as having a low risk of bias if all items adhered to the Cochrane criteria; some concerns were assigned if one or more domains raised some concerns; and a high risk of bias was designated when two or more domains failed to meet the methodological standards^18^.

### Definition of failure

The term “fracture” in the context of restorative failure represents a broad spectrum of defects that differ markedly in their clinical impact. As outlined by the FDI World Dental Federation criteria^19^, the clinical effectiveness of a restoration presenting with a fracture depends on the extent, location, and functional consequence of the defect. Superficial or microcrack formations, such as fine craze lines or glaze fractures, are typically confined to the surface and do not compromise mechanical integrity or marginal adaptation; thus, they are considered clinically acceptable. Similarly, minor chipping or localized material loss that does not affect contact points, occlusal function, or marginal seal may be managed through repair rather than replacement and remains within acceptable clinical performance in case of no signs of biological failure. In contrast, partial or bulk fractures that result in loss of anatomical form, exposure of dentin, or compromise of marginal integrity are deemed clinically unacceptable and represent true restoration failure. Therefore, the clinical effectiveness of a fractured restoration is contingent upon whether the defect remains functionally and structurally stable, rather than the mere presence of a fracture line. In relation to biological failure, since all included teeth were vital, biological failure was defined as any event leading to endodontic treatment, including recurrent caries or radiographic periapical lesions. Notably, marginal staining or discoloration of the restoration without pulp involvement was not considered as a biological failure.

### Effect size

Effect size was calculated using relative risk (RR) with corresponding 95% confidence intervals (CI), considering the number of failures as the event count. Accordingly, the data from each included study were analysed using RevMan 5.3 program (Cochrane Group, London, UK).

### Synthesis methods

Meta-analysis would be conducted if the relevant studies showed sufficient homogeneity after qualitative analysis of the identified data items from each study. In this regard, heterogeneity was evaluated using I^2^, and statistical significance was established as a p-value ≤ 0.05 (Z test).

### Certainty assessment

The overall strength or robustness of the current evidence was assessed using “Grading of Recommendations Assessment, Development and Evaluation” (GRADE). Summary of Findings (SoF) tables were formulated using GRADEpro software.

## Results

**Fig. 1:**
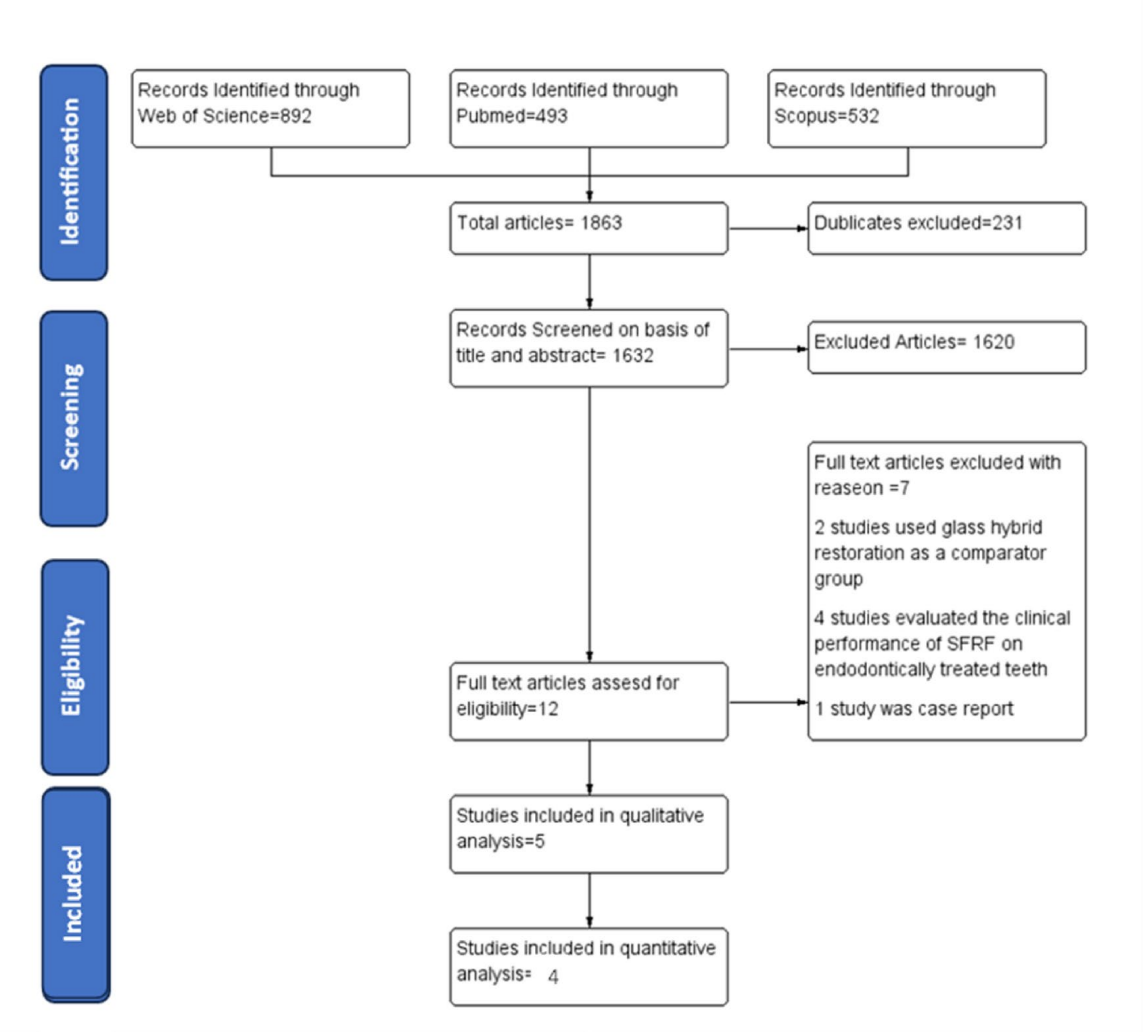
PRISMA Flow diagram of the study screening and selection. PRISMA = Preferred Reporting Items for Systematic Reviews and Meta-Analyses^20^.

After a comprehensive search of the aforementioned online databases, 1,863 articles were identified. Following the removal of duplicates, 1,632 articles remained for title and abstract screening. Based on the applied eligibility criteria, 12 articles were considered eligible for full-text assessment. Ultimately, 5 studies were included in the qualitative analysis, while 4 articles were included in the quantitative analysis (Fig. 1).

### Characteristics of the included studies

The included studies in the current systematic review were published between 2013 and 2025^21–25^. Four of 5 studies reported the number of patients regarding their gender (male and female)^21–23,25^ which were 75 and 154 respectively. Additionally, three of five studies reported the range of patients’ age which was 8–40 years^21,22,24^. Regarding cavity design, three studies reported that the restorations were placed in class II cavities^21–23^, while one study reported that the restorations were placed in extensive MO, or DO, or MOD cavities^24^, and another one study reported that the restorations were placed in only extensive MOD cavities^25^. Regarding study design, four of the five studies were randomized controlled trials with parallel arm design^21–23,25^, while one study was mix between split mouth and parallel arm design^24^. Regarding evaluation criteria, all studies used the criteria of Modified United Stated Public Health Services USPHS criteria. Finally, the total number of treated patients and restored posterior teeth was 274 and 298, respectively, and the follow-up period of the included studies ranged from 12 to 30 months.

Four of the five studies reported using selective enamel etching followed by the application of self-etch or universal adhesive^21–23,25^, while one study didn’t report sufficient details regarding etching protocol^24^. One study reported using G-ænial Bond (GC, Tokyo, Japan), a self-etch adhesive^22^, another study used G-Premio Bond (GC Corporation, Tokyo, Japan), a universal adhesive^23^, one study employed Single Bond Universal Adhesive (3 M Oral Care Deutschland GmbH, Germany)^25^, another used Adper Single Bond (3 M ESPE, USA), an etch-and-rinse adhesive^24^, and one study used G-ænial Bond (GC, Tokyo, Japan) self-etch adhesive for bonding experimental composite, along with Futurabond (DC, VOCO, Cuxhaven, Germany) universal adhesive with dual cure adhesive resin cement for cementation of indirect prepared nanohybrid composite^21^.

Three of the five studies used ever-X Posterior (GC Corporation, Japan) in the experimental group^21,23,25^, while one study used ever-X Flow (GC, Tokyo, Japan)^22^, one study used Ever Stick Net (Fiber) that can be condensed within composite resin^24^. Four of the five studies used nanohybrid conventional composite resin as a comparator group^21–23,25^, while one study used conventional nano-filled composite resin in this regard^24^. Single study has evaluated three groups (SFRC, conventional RBC, and Ribbond-reinforced composite)^25^. Only the SFRC and conventional RBC groups were included in the quantitative synthesis, as the Ribbond group did not meet the inclusion criteria for the present meta-analysis.” Details about characteristics of included studies are presented in Table 1.

**Table 1:** Characteristics of included studies.

Study	Type of study	Number of patients (Male/female)	Number of teeth restored	Average Age (Range)	Follow-up period	Groups	Patient/group	Teeth per group	Tooth type	Cavity type	Evaluation criteria	Etching method	Adhesive used	Placement technique
ElAziz et al.^21^	Randomized controlled trial (1:1)	76 participants (25 males/ 51 females	76	28-31	12 months	1-SFRC (Ever X Posterior, GC, Tokyo, Japan	38	38	–	Class II	USPHS	Selective enamel etching with 35% phosphoric acid gel ( Vococid; VOCO GmbH, Cuxhaven, Germany	G-ænial Bond (GC, Tokyo, Japan (One-component self-etching light-cured adhesive)	Injected
						2-Nanohybrid resin composite (GrandioSo, VOCO, Cuxhaven, Germany						Selective enamel etching with 35% phosphoric acid gel ( Vococid; VOCO GmbH, Cuxhaven, Germany	(Futurabond DC, VOCO, Cuxhaven, Germany + Dual-curing adhesive resin cement (Bifix QM, VOCO, Cuxhaven, Germany)	Cemented
ElAziz et al.^22^		70 (20 male / 50 female)	70	20-40	18 months	1-conventional particulate filler resin composite (PFC) (G-ænial Posterior, GC)	35	35	Premolar and molar teeth	Class II	USPHS	selective enamel etching approach with 37% phosphoric acid gel (Scotchbond, 3 M ESPE, USA) for a duration of 15 s	one-step self-etch bonding agent application (G-ænial Bond, GC)	Incrementally layered
						2-EverX Flow™ (GC, Tokyo, Japan)						selective enamel etching approach with 37% phosphoric acid gel (Scotchbond, 3 M ESPE, USA) for a duration of 15 s	one-step self-etch bonding agent application (G-ænial Bond, GC)	Injected
Salem et al.^23^	Randomized controlled trial (1:1)	36 (9 males/27 females)	36	N/A	12 months	1-G-ænial Sculpt, Nanohybrid composite (GC Corporation, Tokyo, Japan)	18	18	Molar	Class II	USPHS	Selective enamel etching for 15 s using 37% phosphoric acid (Ultradent, South Jordan, USA)	Universal adhesive (G-premio Bond, GC Corporation, Tokyo, Japan)	Incrementally layered
						2-EverX posterior(GC Corporation, Tokyo, Japan)						Selective enamel etching for 15 s using 37% phosphoric acid (Ultradent, South Jordan, USA)	Universal adhesive (G-premio Bond, GC Corporation, Tokyo, Japan)	Injected
Candanet al.^24^	Randomized controlled trial	47 (21 male/ 26 female)	71	8-13	30 months	1-Filtek Supreme (Nanofilled resin composite)	47	35	Molar	Class II and extensive cavities (MO, DO, MOD)	USPHS	N/A	(Adper Single Bond, 3 M ESPE USA)	Incrementally layered
						2-EverStickNet (Fiber)	47	36				N/A	(Adper Single Bond, 3 M ESPE USA)	Condensed
Hamdy et al.^25^	Randomized controlled trial (1:1:1)	45(N/A)	45	N/A	18 months	1- Filtek Z250 XT (3 M, Oral care, USA)	15	15	Molar and premolar	Class II MOD	USPHS	Selective enamel etch with 37 phosphoric acid (Ivoclar, Schaan, Liechtenstein)	Single Bond Universal Adhesive (3 M Oral Care Deutschland GmbH, Germany)	Incrementally layered Injected
						2-EverX Posterior (GC Corporation, Japan)	15	15						
						3-Ribbond-Ultra (Ribbond Inc., Seattle, WA, USA)	15	15						

### Qualitative analysis of the studies

A qualitative analysis of the studies was performed, revealing that all studies reported clinically successful and acceptable restorations in both the experimental and control groups across various clinical parameters according to the USPHS criteria. All studies reported absence of fracture, secondary caries, and loss of anatomic contour (wear). Regarding color match, three of the five studies reported restorations with color mismatch at the end of designed follow-up period in the study. One study reported that 3 restorations had Brave score in the experimental group (SFRC) after 18-month follow-up^22^, One study reported that 12 restorations had a Bravo score in the experimental group after 12-month follow-up, and one study reported that 4 restorations in the experimental group had Bravo score after 18-month follow-up. Regarding absence of marginal discoloration, one study reported that 2 restorations had Bravo score in the experimental group after 12-month follow-up^21^, 2 studies reported that 1 restoration had Brave score in the experimental group after 18 and 30-month follow-up, respectively^22,24^, and one study reported that 2 restorations in the experimental group had Bravo score after 18-month follow-up. Regarding adequate marginal integrity, one study reported that 4 restorations had Bravo score in the experimental group after 12-month follow-up^21^, one study reported that 3 restorations had Bravo score in the experimental group after 18-month follow-up^22^, and one study reported that 1 restoration had Bravo score in the experimental group after 18-month follow-up^25^. Finally, regarding absence of post-operative hypersensitivity, one study reported that 2 restorations had Bravo score in the experimental group after 12-month follow-up^21^. The quantitative data of different clinical parameters extracted from all included studies and are presented in supplementary file 2.

### Analysis of risk of bias of included studies

**Fig. 2:**
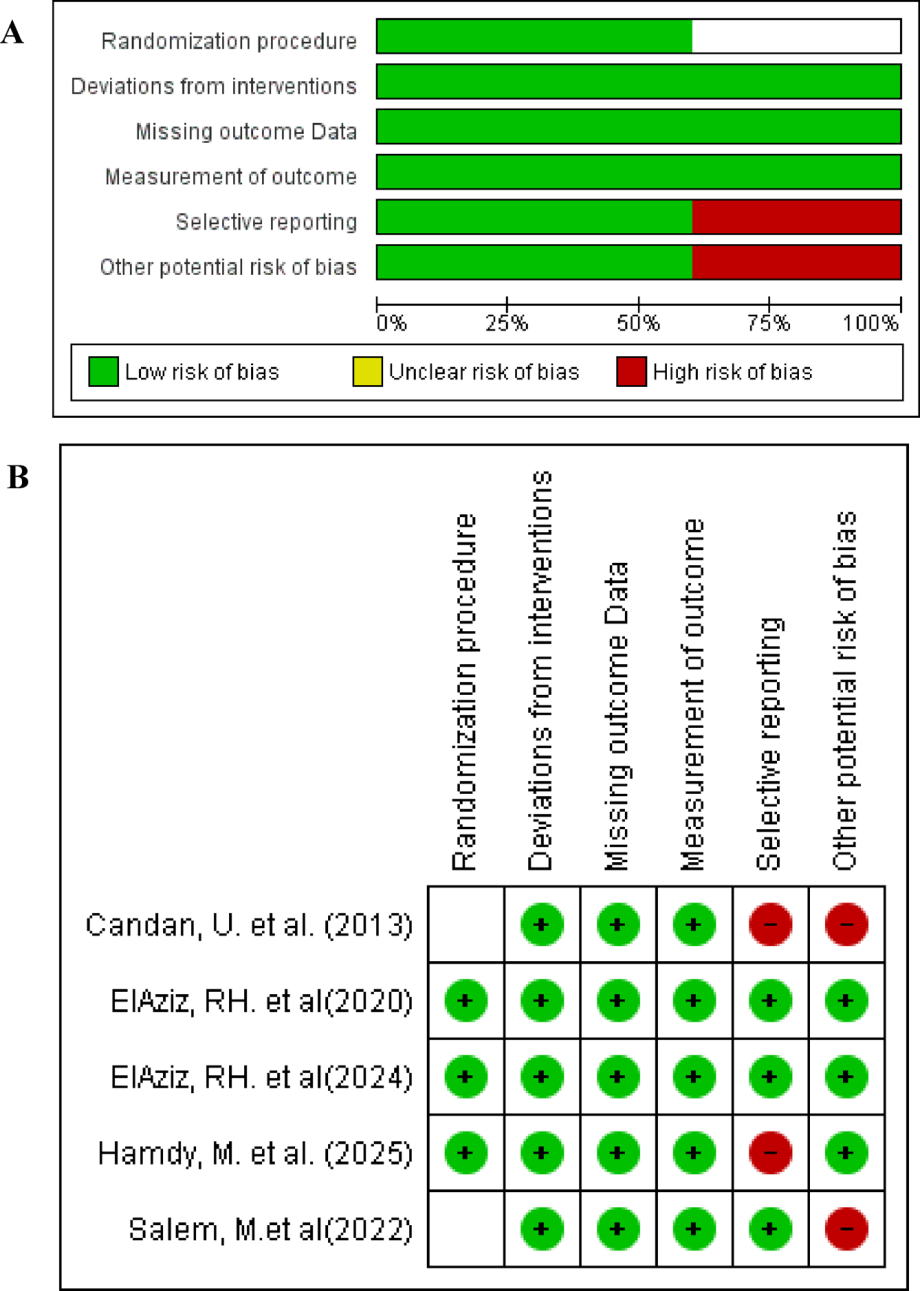
(**A**) Risk of bias graph of included studies. (**B**) Risk of bias summery of included studies.

Risk of bias assessment has been presented in details in Table 2 and illustrated in Figure 2. Two studies showed low risk of bias^21,22^, one study showed some concerns^25^, and two studies showed high risk of bias^23,24^. The most common sources of bias were: Incomplete or selective reporting of outcomes^23–25^, unclear randomization methods^23,24^, and deviations from intended interventions and missing data^23^.

**Table 2:** Risk of bias assessment of included studies.

Study name	Randomization procedure	Deviations from interventions	Missing outcome Data	Measurement of outcome	Selective reporting	Other potential risk of bias	Overall assessment
ElAziz et al.^21^	Low	Low	Low	Low	Low	Low	Low
ElAziz et al.^22^	Low	Low	Low	Low	Low	Low	Low
Salem et al.^23^	Some concerns	Low	Low	Low	Low	High	High
Candan et al.^24^	Some concerns	Low	Low	Low	High	High	High
Hamdy et al.[25]	Low	Low	Low	Low	High	Low	Some concerns

### Synthesis of the results (meta-analysis)

Quantitative analysis was used for four of five studies that were included in qualitative analysis. The study that was omitted from quantitative analysis used everStick NET external fibers instead of ever-X posterior/flow composite in experimental group, unlike the rest of studies^24^. Analysis of the clinical performance of SFRC and RBC was based on 8 different parameters according to modified USPHS criteria including absence of post-operative hypersensitivity, absence of marginal discoloration, absence of fracture, absence of recurrent (secondary) caries, adequate color match, adequate marginal adaptation, adequate surface texture, and adequate anatomical contact. The results were presented as forest plots; where: CI = confidence interval; df = degrees of freedom; M-H = Mantel-Haenszel (fixed-effect model); I^2^ = Higgins I^2^ test; Tau^2^ = Tau square test, Chi^2^ = Chi square test. Meta-analysis for different clinical parameters following modified USPHS criteria revealed that there were no significant differences between SFRCs and conventional nanohybrid RBCs regarding all different clinical parameters. Regarding heterogeneity, color match and marginal adaptation analysis showed considerable level of heterogeneity; (Tau^2^ = 0.05, Chi^2^ = 9.80, df = 2 (*p* = 0.007), I^2^ = 80%), and (Tau^2^ = 0.02, Chi^2^ = 9.92, df = 3 (*p* = 0.02), I^2^ = 70%) respectively, accordingly, random-effect model was selected followed by sensitive analysis for both clinical parameters.

**Fig. 3:**
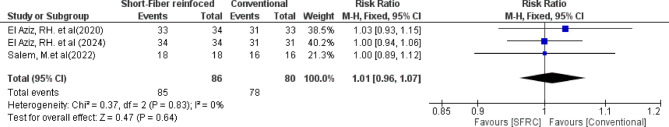
Forest plot for the included studies in terms of absence of post-operative hypersensitivity in restorations.

Regarding absence of post-operative hypersensitivity, the overall relative risk between SFRC and the conventional RBC did not favor either of the two groups (pooled effect size = 1.01, 95% CI= [0.96 to 1.07], *P* = 0.64). Pooled studies were homogenous (Chi-square *P* = 0.83, I-square = 0%) (Fig. 3).

**Fig. 4:**
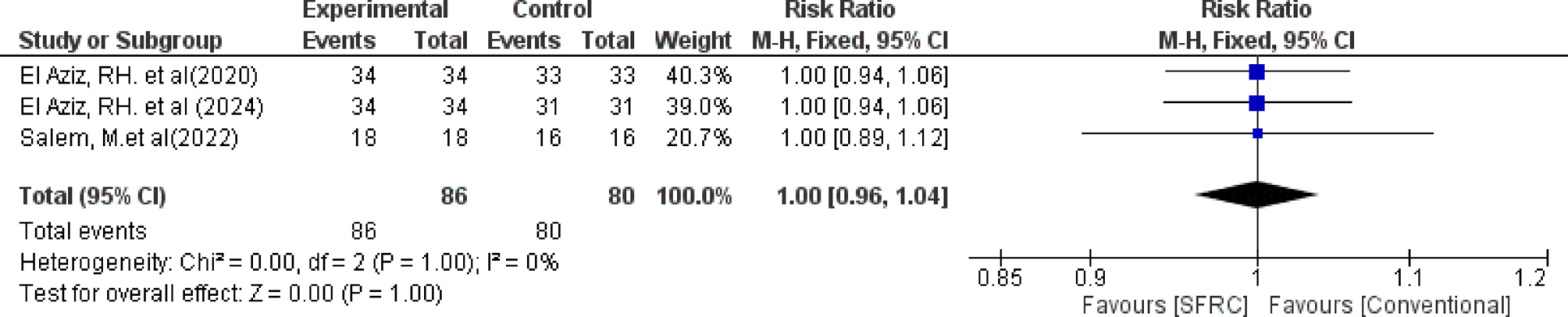
Forest plot for the included studies in terms of adequate surface texture of restorations.

Regarding adequate surface texture, the overall relative risk between the SFRC and the conventional RBC did not favor either of the two groups (pooled effect size = 1.00, 95% CI= [0.96 to 1.04], *P* = 1.00). Pooled studies were homogenous (Chi-square *P* = 1.00, I-square = 0%) (Fig. 4).

**Fig. 5:**
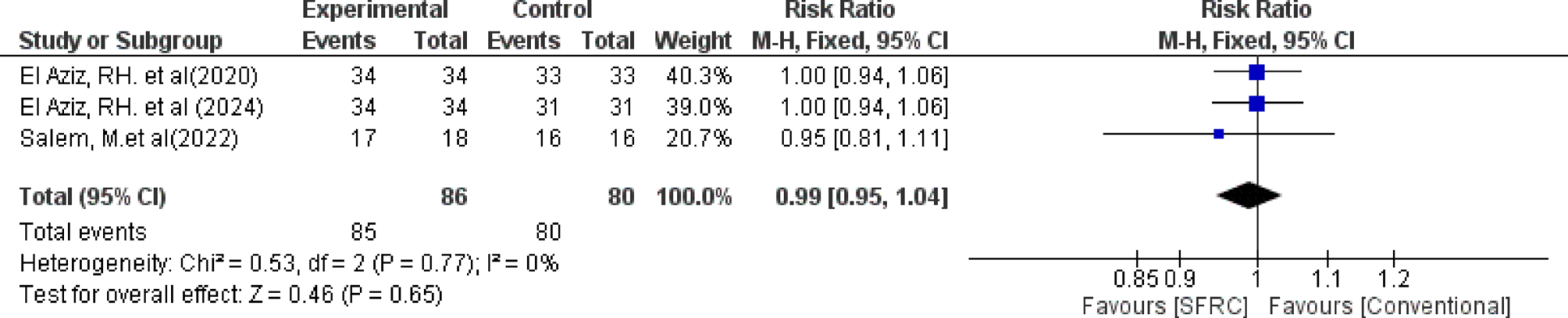
Forest plot for the included studies in terms of adequate anatomical contact of restorations.

Regarding adequate anatomical contact, the overall relative risk between the SFRC and the conventional RBC did not favor either of the two groups (pooled effect size = 0.99, 95% CI= [0.95 to 1.04], *P* = 0.65). Pooled studies were homogenous (Chi-square *P* = 0.77, I-square = 0%) (Fig. 5).

**Fig. 6:**
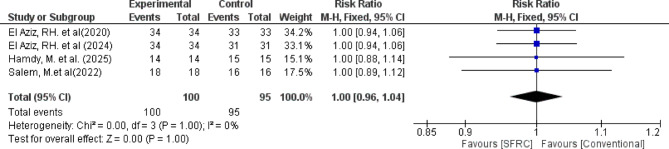
Forest plot for the included studies in terms of absence of recurrent caries around restorations.

Regarding absence of recurrent caries, the overall relative risk between the SFRC and the conventional RBC did not favor either of the two groups (pooled effect size = 1.00, 95% CI= [0.96 to 1.04], *P* = 1.00). Pooled studies were homogenous (Chi-square *P* = 1.00, I-square = 0%) (Fig. 6).

**Fig. 7:**
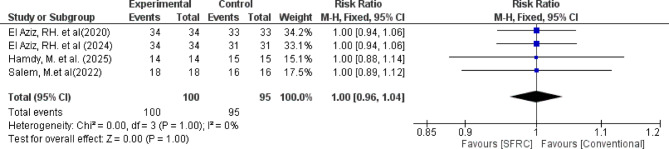
Forest plot for the included studies in terms of absence of fracture of restorations.

Regarding absence of fracture, the overall relative risk between the SFRC and the conventional RBC did not favor either of the two groups (pooled effect size = 1.00, 95% CI= [0.96 to 1.04], *P* = 1.00). Pooled studies were homogenous (Chi-square *P* = 1.00, I-square = 0%) (Fig. 7).

**Fig. 8:**
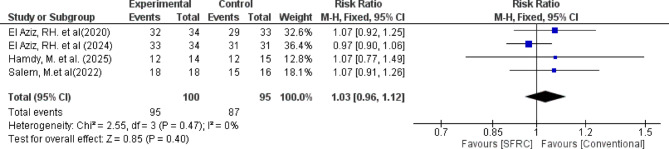
Forest plot for the included studies in terms of absence of marginal discoloration around restorations.

Regarding absence of marginal discoloration, the overall relative risk between the SFRC and the conventional RBC did not favor either of the two groups (pooled effect size = 1.03, 95% CI= [0.96 to 1.12], *P* = 0.40). Pooled studies were homogenous (Chi-square *P* = 0.47, I-square = 0%) (Fig. 8)

**Fig. 9:**
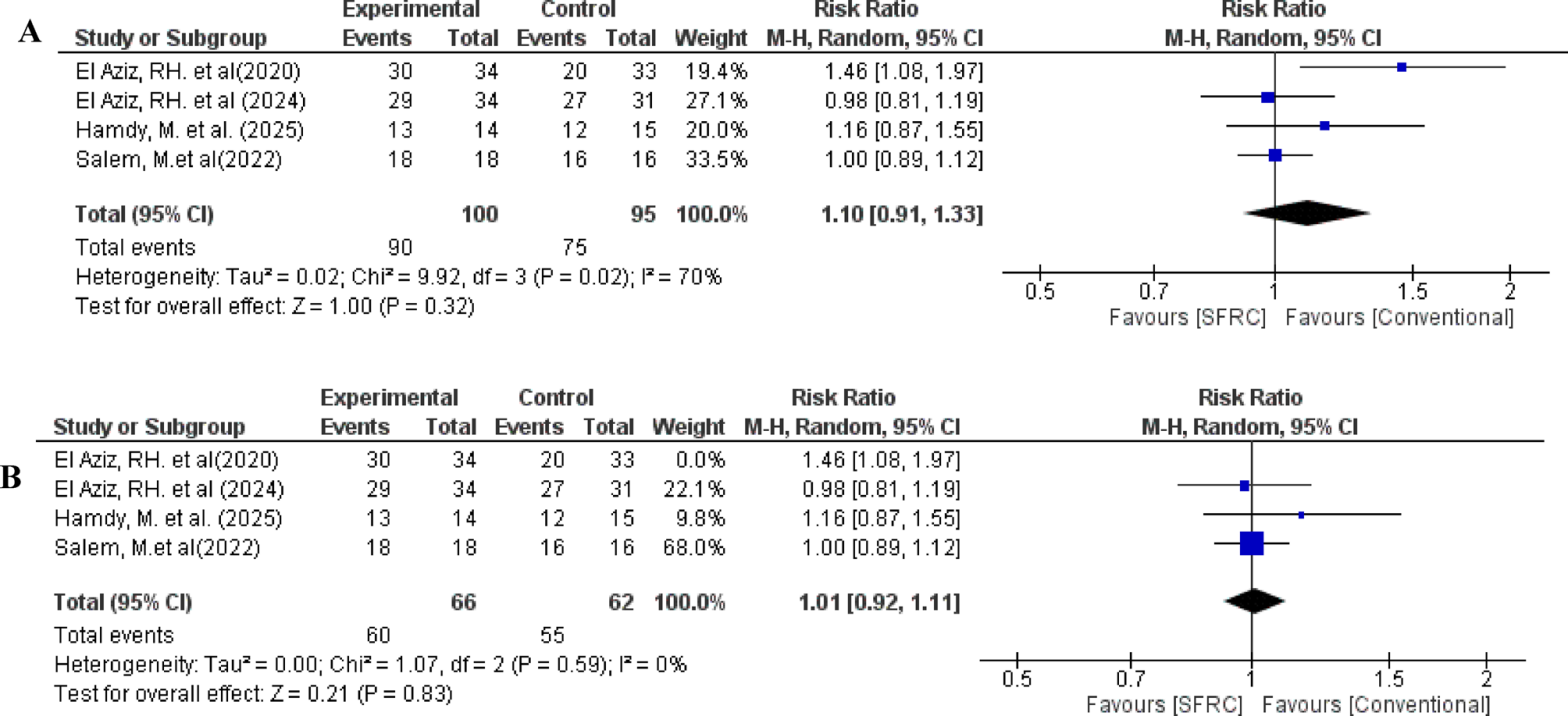
(**A**) Forest plot for the included studies in terms of adequate marginal adaptation. (**B**) Sensitivity analysis to resolve heterogeneity.

Regarding adequate marginal adaptation, the overall relative risk between the SFRC and the conventional RBC did not favor either of the two groups (pooled effect size = 1.10, 95% CI= [0.91 to 1.33], *P* = 0.32). Pooled studies were heterogenous (Chi-square *P* = 0.02, I-square = 70%) (Fig. 9A).

To resolve the heterogeneity, sensitivity analysis was conducted in different scenarios, by excluding one study in each scenario. Heterogeneity was best resolved by excluding the study of El Aziz, RH. et al. 2020 which used nanohybrid indirect composite as a comparator (*P* = 0.59, I-square = 0%). After removing El Aziz, RH. et al. 2020 from the meta-analysis model, the overall risk ratio still doesn’t favor either of two groups (pooled effect size = 1.01, 95% CI= [0.92 to 1.11], *P* = 0.83) (Fig. 9B).

**Fig. 10:**
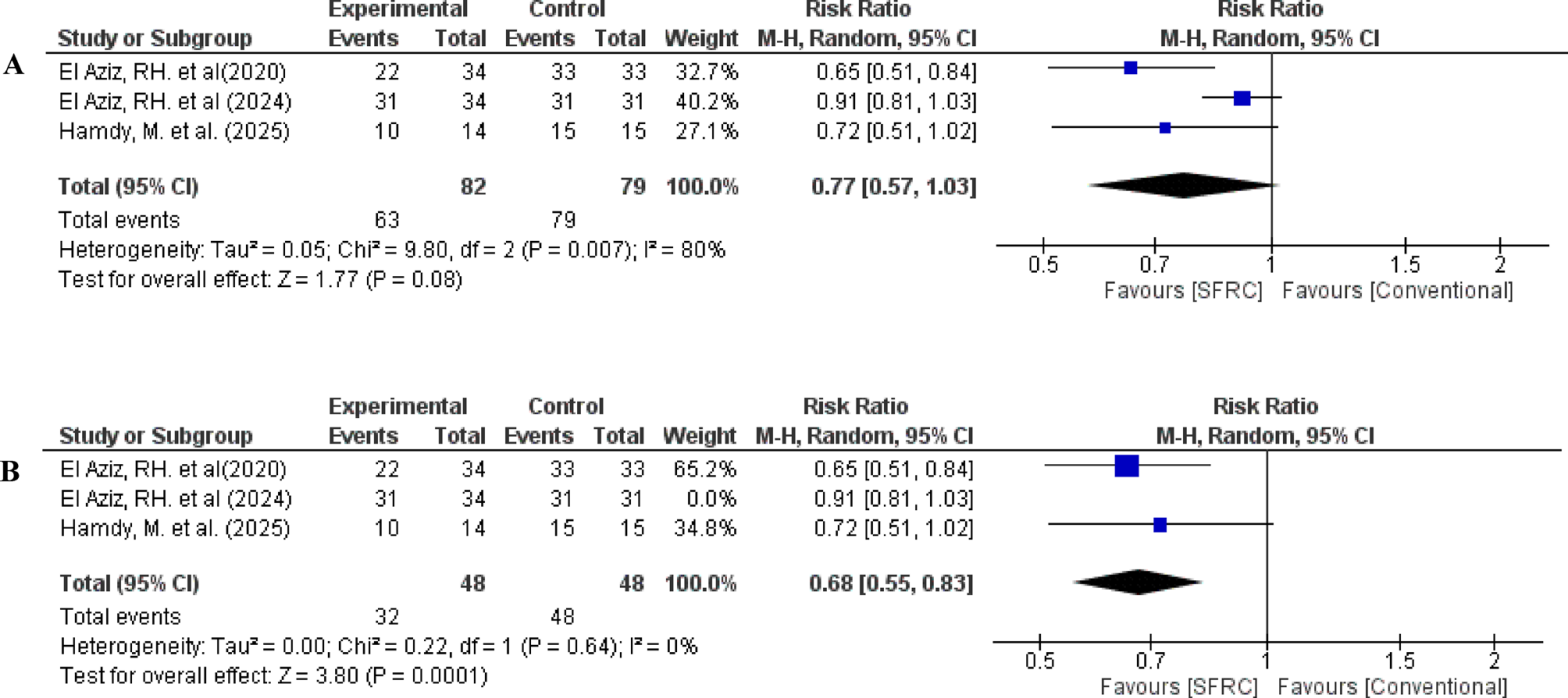
(**A**) Forest plot for the included studies in terms of color match. (**B**) Sensitivity analysis to resolve heterogeneity.

Regarding adequate color match, the overall relative risk between the SFRC and the conventional RBC did not favor either of the two groups (pooled effect size = 0.77, 95% CI= [0.57 to 1.03], *P* = 0.08). Pooled studies were heterogenous (Chi-square *P* = 0.007, I-square = 80%) (Fig. 10A)

To resolve the heterogeneity, a sensitivity analysis was conducted in different scenarios, by excluding one study in each scenario. Heterogeneity was best resolved by excluding the study of El Aziz, RH. et al. 2024(*P* = 0.64, I-square = 0%), which was the only study that used ever-X flow in intervention group. After removing El Aziz, RH. et al. 2024 from the meta-analysis model, the overall risk ratio favoured SFRC group (pooled effect size = 0.68, 95% CI = [0.55 to 0.83], *P* = 0.0001) (Fig. 10B).

#### Subgroup analysis

**Fig. 11:**
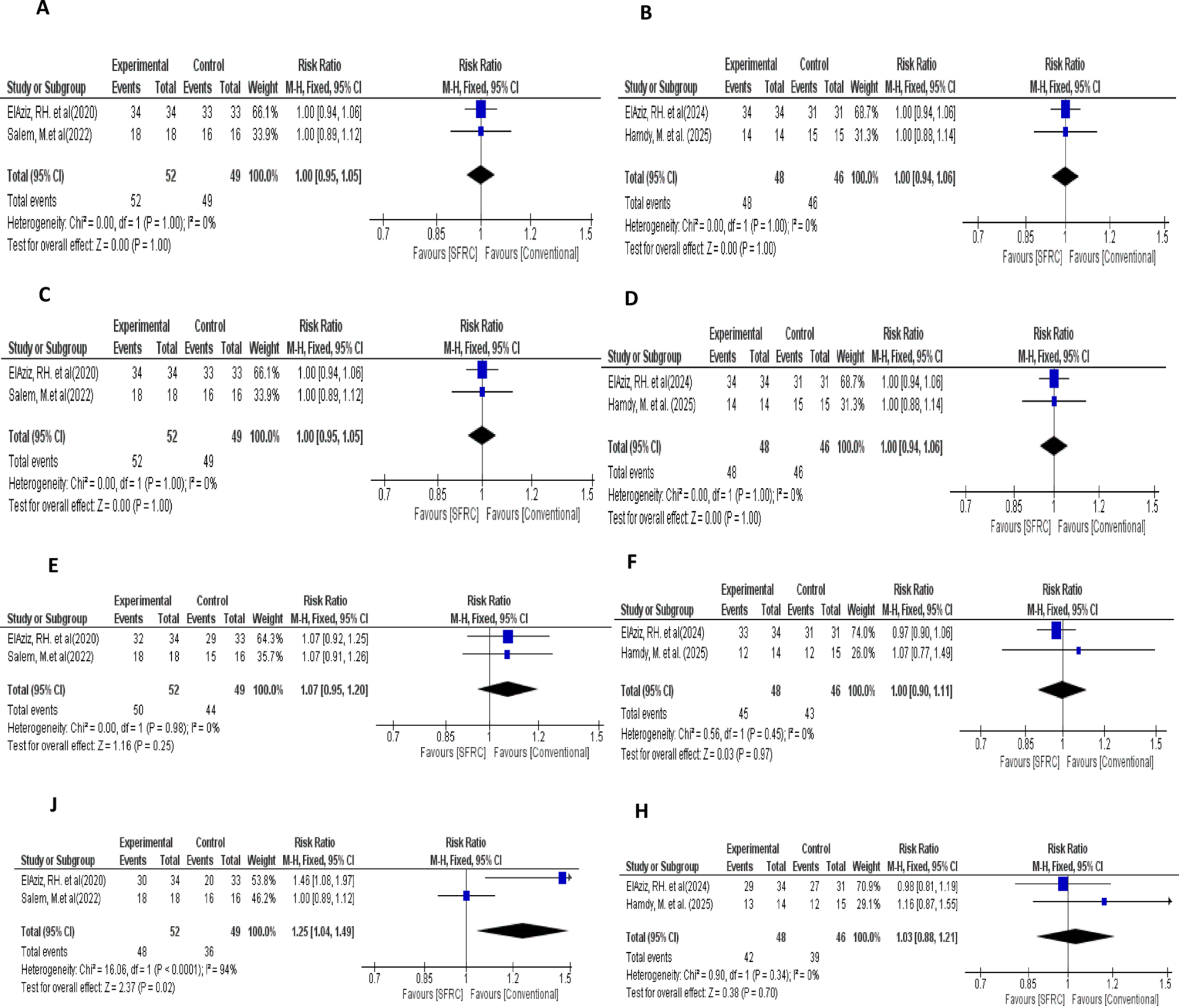
Subgroup analysis of included studies. (**A**) Forest plot of included studies that provided 12-month follow-up in term of absence of recurrent caries. (**B**) Forest plot of included studies that provided 18-month follow-up in term of absence of recurrent caries. (**C**) Forest plot of included studies that provided 12-month follow-up in term of absence of fracture. (**D**) Forest plot of included studies that provided 18-month follow-up in term of absence of fracture. (**E**) Forest plot of included studies that provided 12-monthsfollow-up in term of absence of marginal discoloration. (**F**) Forest plot of included studies that provided 18-month follow-up in term of absence of marginal discoloration. (**J**) Forest plot of included studies that provided 12-month follow-up in term of marginal adaptation. (**H**) Forest plot of included studies that provided 18-month follow-up in term of marginal adaptation.

In relation to follow-up periods, two studies provided 12-month follow-up^21,23^, while the remaining two studies reported 18-month follow-up^22,25^ Accordingly, to manage different follow-up periods, subgroup analysis was conducted. The subgroup analysis at 12- and 18-months follow-up showed no statistically significant difference in outcomes between SFRC and conventional RBC groups across most clinical parameters. (Fig. 11) The consistency of RR ≈ 1.00 and narrow confidence intervals suggest stability of effect (or lack thereof) over time. Although one outcome at 12 months follow-up showed a statistically significant effect favoring the control group (RR 1.25). The extremely high heterogeneity (I² = 94%) undermines confidence in that result. (Fig. 11J) Thus, the aforementioned result justifies the previously conducted sensitivity analysis (Fig. 9B) to resolve the current heterogeneity and improve the confidence of that result.

### Certainty of evidence assessment

Low certainty of evidence was observed regarding most of evaluated outcomes which is ascribed to risk of bias in some studies, inconsistency between studies, and imprecision. The table presenting overall strength of evidence regarding every measured outcome is presented in supplementary file 3.

## Discussion

Resin-based composite restorations are widely regarded as the treatment of choice for small to moderate defects in posterior dentition in different dental literature^8,26–28^. Moreover, another published evidence advocated incremental layering of composite resin for restoring large cavities in posterior dentition^29,30^ Nevertheless, the overall cost of aesthetic posterior restorations remains relatively high; therefore, considerable research attention has been directed toward improving the mechanical properties of these materials to reduce the risk of failure. Several strategies have been implemented to improve the mechanical properties of RBCs varying from using high filler load, changing the size and morphology of filler particles, and utilizing new polymeric matrix to minimize the polymerization shrinkage^8,31–34^. Fiber reinforcement of resin composite restorations is one of promising attempts to improve the mechanical properties of the material. The filler system is reinforced either internally with short discontinuous glass fibers or externally with polyethylene fibers to resist crack propagation within the material^35^. Recently, ever-X posterior composite has been introduced to restorative dentistry consisting of resin matrix, randomly arranged short, discontinued E-glass fibers, and inorganic filler particles. Such a restorative material is biologically inspired, incorporating fibers that function as reinforcing elements, closely mimicking the fibrous structure of dentin. These fibers enhance the material’s ability to absorb and dissipate stresses in a manner similar to natural dentin^36^. Therefore, to achieve both aesthetic and mechanical success in the restoration of extensive posterior cavities, short-fiber reinforced composite can be used in combination with a conventional nanohybrid composite.

Modified USPHS criteria has an expanded use in restorative dentistry as it is described as a standard reliable tool for evaluating direct and indirect restorations. Nevertheless, several published evidence considered modified USPHS criteria as a less reliable, less practical, and highly sensitive evaluation tool compared to FDI criteria^19,37,38^. All included studies in the current review article used modified USPHS criteria for evaluation of the restoration. Mohamed, MH. et al.^25^. adopted the modified USPHS criteria to maintain consistency with prior studies and to ensure that their trial could be appropriately pooled in future systematic reviews.

The current conducted systematic review included 5 studies with a total number of 274 treated patient and 298 restored posterior teeth (molar/premolar) with follow-up periods ranging from 12 to 30 months. The absence of statistically significant differences regarding different clinical parameters between SFRC and conventional RBC indicates that both restorative materials perform consistently across extensive cavities of posterior teeth. Additionally, the findings of the current study suggests that SFRC has comparable mechanical properties to conventional RBC.

Marginal adaptation and discoloration are considered primary indicators when evaluating the clinical performance of restorations. Their outcomes may be affected by several variables, such as the restoration type, cavity dimensions and location, the patient’s oral hygiene practices, and the clinician’s application technique^9,39^. Nevertheless, polymerization shrinkage still the inherent property which is primarily linked to marginal adaptation and discoloration^9,40^. Regarding marginal adaptation and discoloration, no statistically significant differences were detected between SFRC and conventional RBC. The marginal integrity outcomes of SFRC could be attributed to the amount, orientation, and aspect ratio of fibers. Aspect ratio can be defined as a length of the fiber divided by its diameter^25^. The anisotropic material properties are affected by the orientation of fibers, as polymerization shrinkage is controlled in the direction of fibers. Therefore, during polymerization process, the material doesn’t shrink along the fibers’ direction and remain stable horizontally, while the polymer matrix between the fibers can shrink^36,41^. Additionally, the marginal integrity outcome of nanohybrid RBC could be attributed to the dense loading of nano-sized filler particles within the material, thereby reducing the resin friction and polymerization shrinkage^42^. Furthermore, the marginal integrity outcomes justify the absence of secondary caries after different follow-up periods. Notably, regarding marginal adaptation heterogeneity was detected between pooled studies (Chi-square *P* = 0.02, I-square = 70%). This heterogeneity could be attributable to methodological difference between included studies, as one study used indirect nanohybrid composite as a comparator group^21^. Sometimes, differences between studies can be hardly detected visually and requires further complicated statistical testes^43^. Cochran’s Q test is a statistical test commonly used to detect inconsistencies among study results. It does not directly measure the magnitude of heterogeneity, but rather tests the null hypothesis of no heterogeneity by reporting a p-value, typically judged at a 0.05 significance level^43^. Heterogeneity between studies either ascribed to wide confidence interval (95% CI) within the study (within study variability), or studies may vary from each other (between studies variability)^44^. In the current study, heterogeneity is ascribed to both; wide confidence interval within the studies, and methodological differences between them. Furthermore, the varying follow-up periods in the included studies are considered a potential source of heterogeneity. One of the most reported methods to solve heterogeneity is called “subgroup analysis” which depends on categorizing the included studies into two or more distinct groups, and the interaction between subgroup and treatment is examined to determine whether the pooled effect sizes differ significantly across these categories^45^. In subgroup analysis, one outcome (adequate marginal adaptation) at 12-month follow-up showed a statistically significant effect favoring the control group (RR = 1.25) with extremely high heterogeneity (I² = 94%) (Fig. 11J). Thus, sensitivity analysis was conducted by excluding the study that used indirect nanohybrid composite as a comparator from meta-analysis model, hence, heterogeneity was corrected. (*P* = 0.59, I-square = 0%) (Fig. 9B).

Regarding absence of fracture, there was no detected statistically significant difference between the two different restorations. The promising finding of SFRC in this regard could be attributed to the proper stress transfer between the fibers and resin matrix^46^. There is abundance of laboratory evidence that have deeply evaluated and discussed this point. Garoushi, et al.^47,48^ in different laboratory studies evaluated the static load-bearing capacity of direct composite onlay restorations using biomimetic approach compared to bulk-fill and conventional RBCs. SFRCs showed the highest load-bearing capacity compared to other comparator restorations. Ozsevik et al.^49^ reported that, restoring endodontically treated molars with SFRCs covered by conventional RBCs following manufacturer’s instruction provided superior strength that was very close to that of intact teeth and superior to that of the RBC alone and the polyethylene FRC. Alshabib et al.^50^ explained that the crack inhibition mechanism occurs through two distinct processes. First, the fibers can guide the crack along their length; once the crack reaches the end of the short fibers, this disrupts further crack propagation. Second, is the closure force applied on the crack by stretching of fibers causing crack blunting. Accordingly, the aforementioned evidence justifies the current findings of SFRC in this regard.

Notably, a clinical study conducted by van Dijken, WV. et al.^51^ using two different fiber-reinforced composites containing microfibers (Nulite F, and Alert) reported unsatisfactory results in terms of fracture and secondary caries up to 6 years follow-up. Such results could be attributed to the use of fibers with length below the critical fiber length, consequently, there was improper stress transfer from the polymer matrix to the fibers. Van Dijken et al.^51^ reported that there was a clinically observed surface roughness due to exposure of the incorporated fibers. Additionally, they reported that the fracture was observed at the interface between fibers and matrix reflecting the weak bond between matrix and fibers. In contrast, Ever-X posterior composite contains longer fibers, ranging from 0.5 to 1.6 mm. Additionally, the fibers are treated with silane, which promotes strong crosslinking with the polymer matrix; consequently, the fibers can effectively prevent or slow crack propagation.

When considering color stability, SFRC showed no statistically significant difference compared to conventional RBC. Despite that, sensitivity test was performed as color stability results showed high level of heterogeneity I^2^ = 80%. Heterogeneity was best managed in this case by excluding the study that used ever-X flow instead of ever- X posterior composite as an experimental group^22^ (*P* = 0.64, I-square = 0%). After excluding El Aziz, RH. et al.^22^from the meta-analysis model, the overall risk ratio favoured SFRC group (pooled effect size 0.68, 95% CI [0.55 to 0.83], *P* = 0.0001) (Fig. 10B). However sensitivity analysis suggested a possible advantage for RBCs in terms of color match, these findings are based on a limited number of studies and should be interpreted cautiously.

After performing sensitivity analysis, a statistically significant difference in color match was detected between SFRC and conventional RBC as relative risks favoured SFRC. Such a color mismatch might be attributable to the translucency of SFRC due to obtaining short E-glass fiber s which might alter the optical properties of the restorations^52,53^. Accordingly, it is advisable to use opaquer under SFRC or increase the thickness of overlying veneering nanohybrid composite to solve this issue.

Regarding absence of post-operative hypersensitivity, no statistically significant differences were detected between the two restorations. In case of SFRC, the absence of postoperative hypersensitivity is attributable to low polymerization shrinkage of the material, thereby, less stresses could be conducted to cavity margins^54^. Moreover, all included studies used selective enamel etching protocol which has a great influence on this clinical parameter.

Regarding adequate anatomical proximal contact, there were no statistically significant differences detected between the two restorations. It worth mentioning that, one included study used ever-X flow SFRC in class II cavities without proximal coverage with conventional RBC following manufacturer’s recommendation. Such promising findings of ever-X flow regarding anatomical contact and even fracture resistance are in accordance with Lassila et al. findings^55^. They stated that the microscale diameter (6 μm), and length (200–300 μm) of ever-X flow fibers provided superior fracture toughness, and wear resistance compared to other fiber reinforced composites; NovaPro-Fill, Alert, ever X Posterior, and NovaPro-Flow.

Two of five studies used external fibers for resin composite reinforcement by using two different brands which are Ribbond and everstick NET fibers^24,25^. These fibers are arranged in a woven network; therefore, they can distribute the stresses across wide surface area. Ribbond fibers are polyethylene fibers with high elastic modulus and low flexural strength which increase toughness of resin composite and reduce the interfacial stresses along cavity margins^56^. While everstick NET fibers are silanted glass fibres that can resist tensile stresses and act as a crack stopper^24^. Although both types of fibers showed acceptable findings regarding different clinical parameters, there use required expert and skilful operators as they should be impregnated with fluid resin prior to their use to enhance their morphology and clinical effectiveness.

In a systematic review and meta-analysis, a funnel plot is typically recommended to assess publication bias or small-study effects. However, in this case the funnel plot cannot be conducted due to few numbers of included studies (< 10) which may give misleading results^57^.

Certainty or quality of evidence can be defined as a reflection of confidence that the true effects lie on one side of a specified threshold or within a specific range. Certainty of evidence can be rated as high, moderate, low, or very low. High certainty means being very confident that the true effect lies close to estimate effect. Moderate certainty means being confident that true effect lies close to estimate effect but with some substantial differences. While, in case of low certainty, it means being confident that the true effect may be substantially different from effect estimate. Accordingly, The Grading of Recommendations Assessment, Development, and Evaluation (GRADE) approach provides a framework for rating certainty or quality of systematic review, minimizing risk of bias, and making the strength of evidence and study recommendations trustworthy^58^. After certainty assessment of the current systematic review, it was found that the robustness of evidence is low. The main factors contributing to downgrading were risk of bias, imprecision, and inconsistency across the included studies. Risk of bias was mainly attributed to limited reporting of randomization procedures, potential deviations from intended interventions, and selective data reporting in some studies. Imprecision reflected the relatively wide confidence intervals and small sample sizes, which reduced confidence in the pooled effect estimates. Inconsistency was noted particularly for marginal adaptation and color match, where moderate to substantial heterogeneity (I² = 70–80%) was observed.

### Limitations

The overall methodological quality of the included trials was variable, with several studies lacking clear descriptions of standardization protocol and outcome reporting. These limitations may introduce performance or detection bias, potentially influencing subjective clinical parameters such as marginal adaptation and color match. Furthermore, due to the limited number of included studies, a formal assessment of publication bias (e.g., funnel plot or Egger’s test) was not feasible, which restricts our ability to rule out selective publication of positive results. Despite these limitations, the overall evidence suggests that SFRCs perform comparably to conventional RBCs in terms of fracture resistance, marginal integrity, color stability, and biological outcomes such as absence of secondary caries and postoperative hypersensitivity. The moderate certainty of evidence for surface texture supports the reliability of this finding, whereas other outcomes with low or very low certainty should be interpreted cautiously. Further high-quality, multicenter randomized controlled trials with standardized outcome measures and longer follow-up are warranted to strengthen the evidence base and improve the precision of future meta-analyses.

## Conclusion

Based on the currently available clinical data, there is low-quality evidence indicating that short fiber-reinforced composite restorations exhibit comparable clinical performance to conventional resin-based composites in posterior cavities. However, given the limited number of studies, small sample sizes, and relatively short follow-up durations, these findings should be interpreted with caution. Further well-designed, long-term randomized controlled trials are required to validate these preliminary results and strengthen the evidence base.

## Supplementary Information

Below is the link to the electronic supplementary material.

Supplementary Material 1

Supplementary Material 2

Supplementary Material 3

Supplementary Material 4

## Data Availability

All the data are included in supplementary files.
