# Supplementary material for: Clinical performance of short fiber-reinforced composite resin restoration in large posterior cavities: a systematic review and meta-analysis
Source: Sci Rep. 2025 Dec 22;15:44334. doi: 10.1038/s41598-025-31441-z (PMC12727727; doi:10.1038/s41598-025-31441-z)
Supplement: Supplementary file 1 — Supplementary Material 1 [file 41598_2025_31441_MOESM1_ESM.docx]

**Search strategy in different databases**

| **Database** | **Search keywords** |
| --- | --- |
| **PubMed** | ("Composite Resins"[MeSH] OR "Fiber-Reinforced Composite"[tiab] OR "Short Fiber Composite"[tiab] OR "Ever X Posterior"[tiab] OR "Ever X Flow"[tiab]) AND ("Dental Restoration, Permanent"[MeSH] OR "Composite Resin Restoration"[tiab] OR "Nanohybrid Composite Resin"[tiab] OR "Particulate Filler Composite"[tiab]) AND ("Clinical Performance"[tiab] OR "Clinical Longevity"[tiab] OR "Clinical Behaviour"[tiab]) AND ("Posterior Teeth"[MeSH] OR "Posterior Cavity"[tiab] OR "Molar"[MeSH] OR "Premolar"[MeSH]) |
| **Web of science** | ALL=("Fiber reinforced composite resin") OR ALL=("Short fiber reinforced composite resin") OR ALL=("E-glass fibers") OR ALL=("Ever X Posterior") OR ALL=("Ever X Flow") AND ALL=("Conventional composite resin restoration") OR ALL=("Nanohybrid composite resin") OR ALL=("Particulate filler composite") OR ALL=("Composite resin restoration") AND ALL=("Clinical performance") OR ALL=("Clinical longevity") OR ALL=("Clinical behaviour") AND ALL=("Posterior teeth") OR ALL=("Posterior cavities") OR ALL=("Posterior cavity") OR ALL=("Molar") OR ALL=("Premolar") |
| **Scopus** | TITLE-ABS-KEY("fiber reinforced composite resin" OR "short fiber reinforced composite resin" OR "fiber reinforced composite" OR "e-glass fibers" OR "Ever X Posterior" OR "Ever X Flow" OR "composite resin restoration" OR "particulate filler composite" OR "conventional composite resin" OR "nanohybrid composite resin" OR "clinical performance" OR "clinical longevity" OR "clinical behaviour" OR "posterior teeth" OR "posterior tooth" OR "posterior cavities" OR "posterior cavity" OR "molar" OR "premolar") |
