# Supplementary material for: Clinical performance of short fiber-reinforced composite resin restoration in large posterior cavities: a systematic review and meta-analysis
Source: Sci Rep. 2025 Dec 22;15:44334. doi: 10.1038/s41598-025-31441-z (PMC12727727; doi:10.1038/s41598-025-31441-z)
Supplement: Supplementary file 2 — Supplementary Material 2 [file 41598_2025_31441_MOESM2_ESM.docx]

**Quantitative data of different clinical parameters of included studies**

|  | **Absence of Postoperative**  **hypersensitivity**  **(Alpha/Bravo)** | **Adequate Color match**  **(Alpha/Bravo)** | **Adequate Marginal integrity**  **(Alpha/Bravo)** | **Absence of Marginal discoloration**  **(Alpha/Bravo)** | **Absence of secondary caries**  **(Alpha/Bravo)** | **Adequate Surface texture**  **(Alpha/Bravo)** | **Absence of fracture**  **(Alpha/Bravo)** | **Anatomical contact (Absence of wear)** **(Alpha/Bravo)** |
| --- | --- | --- | --- | --- | --- | --- | --- | --- |
| **ElAziz, RH. et al**  **(2020)** | CC:  Total =33  Event=31  SFRC:  Total =34  Event=33 | CC:  Total =33  Event=33  SFRC:  Total =34  Event=22 | CC:  Total =33  Event=20  SFRC:  Total =34  Event=30 | CC:  Total =33  Event=29  SFRC:  Total =34  Event=32 | CC:  Total =33  Event=33  SFRC:  Total =34  Event=34 | CC:  Total =33  Event=33  SFRC:  Total =34  Event=34 | CC:  Total =33  Event=33  SFRC:  Total =34  Event=34 | CC:  Total =33  Event=33  SFRC:  Total =34  Event=34 |
| **ElAziz, RH. et al**  **(2024)** | CC:  Total =31  Event=31  SFRC:  Total =34  Event=34 | CC:  Total =31  Event=31  SFRC:  Total =34  Event=31 | CC:  Total =31  Event=27  SFRC:  Total =34  Event=29 | CC:  Total =31  Event=31  SFRC:  Total =34  Event=33 | CC:  Total =31  Event=31  SFRC:  Total =34  Event=34 | CC:  Total =31  Event=31  SFRC:  Total =34  Event=34 | CC:  Total =31  Event=31  SFRC:  Total =34  Event=34 | CC:  Total =31  Event=31  SFRC:  Total =34  Event=34 |
| **Candan, U. et al. (2013)** | CC:  Total =35  Event=35  SFRC:  Total =36  Event=36 | CC:  Total =35  Event=33  SFRC:  Total =36  Event=35 | CC:  Total =35  Event=33  SFRC:  Total =36  Event=34 | CC:  Total =35  Event=33  SFRC:  Total =36  Event=35 | CC:  Total =35  Event=35  SFRC:  Total =36  Event=36 | CC:  Total =35  Event=34  SFRC:  Total =36  Event=35 | N/R | CC:  Total =35  Event=35  SFRC:  Total =36  Event=36 |
| **Salem, M.et al**  **(2022)** | CC:  Total =16  Event=16  SFRC:  Total =18  Event=18 | N/R | CC:  Total =16  Event=16  SFRC:  Total =18  Event=18 | CC:  Total =16  Event=15  SFRC:  Total =18  Event=18 | CC:  Total =16  Event=16  SFRC:  Total =18  Event=18 | CC:  Total =16  Event=16  SFRC:  Total =18  Event=18 | CC:  Total =16  Event=16  SFRC:  Total =18  Event=18 | CC:  Total =16  Event=16  SFRC:  Total =18  Event=17 |
| **Hamdy, M. et al. (2025)** | N/R | CC:  Total =15  Event=15  SFRC:  Total =14  Event=10 | CC:  Total =15  Event=12  SFRC:  Total =14  Event=13 | CC:  Total =15  Event=12  SFRC:  Total =14  Event=12 | CC:  Total =15  Event=15  SFRC:  Total =14  Event=14 | N/R | CC:  Total =15  Event=15  SFRC:  Total =14  Event=14 | N/R |

**CC**: Conventional composite. **SFRC**: short fiber-reinforced composite. **N/R**: Not reported

**Alpha** = ideal; **Bravo** = clinically acceptable; **Charlie** = failure. Success was defined as Alpha or Bravo scores
