# Supplementary material for: Clinical performance of short fiber-reinforced composite resin restoration in large posterior cavities: a systematic review and meta-analysis
Source: Sci Rep. 2025 Dec 22;15:44334. doi: 10.1038/s41598-025-31441-z (PMC12727727; doi:10.1038/s41598-025-31441-z)
Supplement: Supplementary file 3 — Supplementary Material 3 [file 41598_2025_31441_MOESM3_ESM.docx]

**Author(s):** Kareem Hamdi

**Question:** [SFRC] compared to [conventional] for [extensive cavities]

**Setting:**

**Bibliography:**

| **Certainty assessment** | | | | | | | **№ of patients** | | **Effect** | | **Certainty** | **Importance** |
| --- | --- | --- | --- | --- | --- | --- | --- | --- | --- | --- | --- | --- |
| **№ of studies** | **Study design** | **Risk of bias** | **Inconsistency** | **Indirectness** | **Imprecision** | **Other considerations** | **[SFRC]** | **[conventional]** | **Relative (95% CI)** | **Absolute (95% CI)** |  |  |
| **Absence of Fracture** | | | | | | | | | | | | |
| 4 | randomised trials | serious^a^ | not serious | not serious | serious^b^ | none | 100/100 (100.0%) | 95/95 (100.0%) | **RR 1.00** (0.96 to 1.04) | **0 fewer per 1,000** (from 40 fewer to 40 more) | ⨁⨁◯◯ Low^a,b^ |  |
| **Absence of Post operative hypersensitivity** | | | | | | | | | | | | |
| 3 | randomised trials | serious^a^ | not serious | not serious | serious^b^ | none | 85/86 (98.8%) | 78/80 (97.5%) | **RR 1.01** (0.96 to 1.07) | **10 more per 1,000** (from 39 fewer to 68 more) | ⨁⨁◯◯ Low^a,b^ |  |
| **Adequate surface texture** | | | | | | | | | | | | |
| 3 | randomised trials | not serious^a^ | not serious | not serious | serious^b^ | none | 86/86 (100.0%) | 80/80 (100.0%) | **RR 1.00** (0.96 to 1.04) | **0 fewer per 1,000** (from 40 fewer to 40 more) | ⨁⨁⨁◯ Moderate^a,b^ |  |
| **Adequate anatomical contact** | | | | | | | | | | | | |
| 3 | randomised trials | serious^a^ | not serious | not serious | serious^b^ | none | 85/86 (98.8%) | 80/80 (100.0%) | **RR 0.99** (0.95 to 1.04) | **10 fewer per 1,000** (from 50 fewer to 40 more) | ⨁⨁◯◯ Low^a,b^ |  |
| **Absence of recurrent caries** | | | | | | | | | | | | |
| 4 | randomised trials | serious^a^ | not serious | not serious | serious^b^ | none | 100/100 (100.0%) | 95/95 (100.0%) | **RR 1.00** (0.96 to 1.04) | **0 fewer per 1,000** (from 40 fewer to 40 more) | ⨁⨁◯◯ Low^a,b^ |  |
| **Absence of marginal discoloration** | | | | | | | | | | | | |
| 4 | randomised trials | serious^a^ | not serious | not serious | serious^b^ | none | 95/100 (95.0%) | 87/95 (91.6%) | **RR 1.03** (0.96 to 1.12) | **27 more per 1,000** (from 37 fewer to 110 more) | ⨁⨁◯◯ Low^a,b^ |  |
| **Marginal adaptation** | | | | | | | | | | | | |
| 4 | randomised trials | serious^a^ | serious^c^ | not serious | serious^b^ | none | 90/100 (90.0%) | 75/95 (78.9%) | **RR 1.10** (0.91 to 1.33) | **79 more per 1,000** (from 71 fewer to 261 more) | ⨁◯◯◯ Very low^a,b,c^ |  |
| **Prescence of color match** | | | | | | | | | | | | |
| 3 | randomised trials | not serious | serious^d^ | not serious | serious^b^ | none | 63/82 (76.8%) | 79/79 (100.0%) | **RR 0.77** (0.57 to 1.03) | **230 fewer per 1,000** (from 430 fewer to 30 more) | ⨁⨁◯◯ Low^b,d^ |  |

**CI:** confidence interval; **RR:** risk ratio

#### Explanations

a. Out of 4 pooled studies, 1 showed high risk of bias, while another 1 study showed moderate risk of bias

b. Relatively wide confidence interval CI

c. Pooled studies were heterogenous (Chi-square P=0.02, I-square=70%).

d. Pooled studies were heterogenous (Chi-square P=0.007, I-square=80%).
